# Effect of Case-Based Small-Group Learning on Care Workers’ Emergency Coping Abilities

**DOI:** 10.3390/ijerph182111458

**Published:** 2021-10-30

**Authors:** Soon-Ok Kim

**Affiliations:** Department of Nursing, Shinhan University, Uijeongbu 11340, Korea; kso6210@hanmail.net; Tel.: +82-31-870-0472; Fax: +82-31-870-1719

**Keywords:** case-based learning, small-group, emergencies, coping abilities, care workers

## Abstract

This study aimed to develop and implement an emergency coping education program using a case-based small-group learning method and verify its effect on care workers’ emergency coping abilities. The study was conducted with 72 care workers in older adult care facilities and home care centers. Using a nonequivalent control group pretest–posttest design, 36 participants were assigned to each group (i.e., experimental and control groups). The collected data were analyzed through χ^²^-test and independent t-test using SPSS for Windows, version 25.0. Compared to the control group, a statistically significant increase in knowledge and performance levels in emergencies, emergency coping abilities, self-efficacy in coping with emergencies, and confidence in communication was observed in the experimental group. This study was able to verify the effectiveness of the emergency coping education program in care workers and recommends its use. To maximize the learning effects of educational programs, future research should develop and apply programs that incorporate simulation education.

## 1. Introduction

### 1.1. Background

By 2040, South Korea may become one of the top three OECD members with the largest older adult population [1], and the proportion of older adults is expected to increase by 20.3 and 37% in 2025 and 2045, respectively, domestically [2]. Moreover, owing to a general reality that older adults experience deterioration in their physical, mental, and social health, the general trend is for them to be a risk group health wise. Against this backdrop, and as a response to the increase in geriatric diseases and medical expenses, the South Korean government implemented a long-term care (LTC) insurance system for older adults in 2008. By the end of December 2019, 772,206 South Korean older adults had already been assessed for becoming LTC service (LTCS) recipients, which provides the benefit, for instance, of accessing nursing services [3].

Older adults have a high risk of exposure to emergencies owing to their higher morbidity rate associated with physical, psychological, and social hypofunctions. Approximately 65% of these LTCS recipients are over the age of 80 years [4], many are frail [5], experience an average of 3–4 chronic diseases (e.g., hypertension at 60.3%, dementia at 57.2%, and diabetes at 29.3%) [4], and the proportion of frail older adults is four times higher among LTCS recipients than among older adults in the community [5], and more than 60% have cognitive and physical comorbid disorders [6]. According to Lemoyne et al., these conditions increase the incidence of falls and medical emergencies (e.g., infection and respiratory, cardiovascular, central nervous system, or cognitive disorders) in LTCS recipients [7].

Care workers are key frontline workers who provide care services for older adults most of the time in the field of LTCS. Since emergencies can occur anytime and without notice, in emergencies involving LTCS recipients, it is more likely for care workers to be the first responders [8]. When exerting such roles, being proficient in emergency coping skills could save lives and prevent further accidents. O’Neill et al. recommended that all workers in organizations that deal with high-risk groups for emergencies should be able to find and cope with an emergency at an early stage and be aware of appropriate health management strategies to prevent emergencies [9]. In South Korea, the staffing standards for LTCS delivery in an older adult care facility (OACF) require the availability of at least one care worker for every 2.5 older adults and at least 15 care workers for a home care center (HCC) with more than 30 residents. Among the total workforce of 492,132 people in the LTC insurance system for older adults of South Korea, care workers account for around 90% (444,525) [10]. Given that OACF care workers in South Korea often work two or three shifts, these professionals account for the largest share (87.1%) of employees on duty in the LTC insurance system [11], and they solely provide care services at home for older adults. Therefore, OACF or HCC care workers are highly likely to be present during an emergency among LTCS recipients.

However, the emergency coping ability of care workers at both OACFs and HCCs has been rated low. This aspect is shown in a study by Kim and Kim [12], which reported an average of 74.26 points for care workers at OACFs and 68.02 for those at HCCs. In a study by Kim [13], the coping ability was 60.57 points for OACFs and 57.53 for HCCs. Furthermore, the performance rate of first aid procedures by care workers has been reported to be as low as 30.1%, mainly (77.9%) owing to lack of confidence [14], while their scores for emergency knowledge were lower than 60 points (56.7 and 47.9 points) [14]. This cited study suggests the need to improve the emergency coping abilities of these professionals. According to a study by Lim [15], confidence in performing first aid is a precedent for the practical implementation of these procedures, denoting that enhancing emergency knowledge is the most important prerequisite for the correct performance of these potentially life-saving processes.

Concurring with these past results on care workers’ low emergency coping abilities and the need to improve their first aid performance, a study showed that 98.6 and 96.4% of care workers at OACFs and HCCs, respectively, desired to receive first aid training, indicating a high demand for emergency coping ability training [13]. Accordingly, Lim and Lee [16] insisted that continuous education and training should be provided to improve care workers’ practical performance and to maintain their professionalism. Moreover, well-organized education for care workers can reinforce not only their knowledge about older adults but also the capability to cope with emergencies or situational problems related to older adult care [17]. However, the current lack of refresher training or mandatory occupational training for care workers in South Korea makes it difficult for them to receive emergency training [18]. Additionally, the only training on the topic currently provided for care workers in the country is a 3 h class, which is a part of the basic professional education for caring for patients with dementia [13]. For these reasons, each long-term care facility tries to enhance the emergency coping ability of their care workers by providing them with external and/or internal emergency education sessions [11,19]. Nevertheless, since it is hard to assure the quality of internal education and for small-scale facilities to perform their own education programs, there are various limitations in the implementation of high-quality education on emergency coping for care workers in South Korea.

Care workers are non-medical practitioners and cannot provide medical treatment during emergencies. However, some non-medical practitioners are required to receive emergency education, when necessary, in South Korea. For example, it is mandatory for those who commonly encounter emergencies during their duties (e.g., police officers, fire fighters, rescue workers, and industrial workers) to receive first aid [20]. Moreover, the standard textbook for care workers in South Korea stipulates that they should take suitable measures when faced with an emergency, from calling telephone helplines or emergency services to providing appropriate first aid care. Additionally, these professionals should not stop delivering first aid care until a medical professional arrives to manage the situation [21]. Hence, in a scenario where regular emergency education is lacking, it seems essential to develop and provide an emergency education program for care workers, as it may serve to improve their emergency coping ability. Notwithstanding, since the mean age of care workers ranges from 40–60 years and their educational level differs widely, there is a need to seek proper education methods according to the sociodemographic characteristics of these professionals[13]. Moreover, considering that emergency education should focus on enhancing performance, it is urgently required to develop and apply practice-oriented education [22,23]. To date, many issues have been raised regarding the lack of systematic, lecture-oriented education and the absence of proper education materials, which should be improved in future education programs for care workers [18].

Against this backdrop comes case-based learning (CBL), which is an educational method in which small learner groups acquire problem-solving knowledge and skills through discussion, feedback, and self-reflection, thus promoting the application of theoretical knowledge in practical settings [24]. CBL is regarded as a highly effective educational method. The learning processes are grounded on real-life cases, allowing learners to internalize knowledge and experiences and enhance their skills in a given context [24]. To date, in South Korea, care workers have already availed of various CBL programs on numerous themes (e.g., fall prevention, diaper hygiene nursing, eating behavior coping skills, and basic life support education) [23]. In other countries, these workers have participated in CBL programs on stroke [25] and psychological education [26].

However, there have been no studies verifying CBL-based education to promote emergency coping ability. Therefore, this study aimed to determine whether an education program that combines CBL with practical training using a simulator—developed based on a previous study reporting that individual practical training is effective in enhancing clinical performance [27]—can serve as a teaching method to promote emergency coping ability in care workers and whether such a program impacts their levels of performance in emergencies.

### 1.2. Purpose

This study aimed to develop and implement an emergency coping education program using a case-based small-group learning method for care workers and verify its effect on participants’ emergency coping abilities. Specifically, it is intended to analyze the changes in care workers’ level of knowledge and performance in emergencies at posttest.

### 1.3. Research Hypotheses

(1) The level of knowledge and performance in emergencies will be higher in the experimental group (EG; i.e., care workers who underwent the emergency coping education program) than the control group (CG).
(2) Emergency coping abilities will be higher in the EG than in the CG.
(3) Self-efficacy in coping with emergencies will be higher in the EG than in the CG.
(4) Confidence in communication will be higher in the EG than in the CG.

## 2. Method

### 2.1. Study Design

This was a nonequivalent CG pretest-posttest study.

### 2.2. Study Participants

Through convenience sampling, care workers working at OACFs and HCCs in S City and its metropolitan area were recruited. After receiving information about study purposes and procedures, all the participants provided written informed consent. To prevent the diffusion of the effect of the experiment, the participants in the EG were care workers employed at OACFs and HCCs in S City. The participants in the CG were those working at various facilities and centers related to older adult care in the metropolitan area of S City. These procedures were carried out based on a study that also targeted older adults in long-term care facilities. The researchers used convenience sampling to recruit people who were admitted to different facilities, as this process helps to minimize the spread of the effects of the experiment in long-term care facilities that have older adults with similar characteristics [28].

The sample size was estimated using G*Power, version 3.1.9.4 [29]. With a significance level (α) = 0.05, a power (1 − β) = 0.90, and an effect size for the independent sample *t*-test (d) = 0.80, the minimum sample size for each group was 34 people, totalizing 68 participants. Considering the dropout rate, 76 people were recruited, of which 38 were allocated to each group (i.e., EG and CG). Nonetheless, the data of two care workers were deleted in the EG (i.e., they missed the training more than twice) and two in the CG (i.e., they only attended 10 h of the program owing to personal reasons). Hence, the data from 72 study participants (36 to each the EG and CG) was used in the analyses.

### 2.3. Measurement Tools

#### 2.3.1. Level of Knowledge and Performance in Emergencies in Long-Term Care Facilities

In another study, to evaluate the level of knowledge and performance of early childhood teachers for different emergency situations, Cho et al. developed a 35-item tool that focused on the 10 most frequently occurring safety accidents and emergency accidents in daily life in childcare facilities [30]. For this study, 20 items from this aforementioned tool were selected and modified (for research purposes) that frequently occurred within the context of older adult care.

After final revisions and supplementation, the modified scale was reviewed, and its content validity analyzed by a group of experts (i.e., three nursing professors, two nursing team supervisors at OACFs, and two HCC directors). The modified 20-item tool is reported on a 4-point Likert scale, with total scores ranging from 20–80 points, and higher scores indicating advanced levels of knowledge and performance in emergencies. In Cho et al.’s study, the Cronbach’s α for the level of knowledge and level of practice subscales were 0.92 and 0.88, respectively [30]; in this study, they were 0.86 and 0.87, respectively.

#### 2.3.2. Emergency Coping Ability

In this study, the emergency coping ability scale developed by Hwang and Lee [31] was used. This 20-item tool is reported on a 5-point Likert scale, with total scores ranging from 20–100 points, and higher scores indicating advanced levels of emergency coping ability. In Hwang and Lee’s study, the Cronbach’s α was 0.96 [31]; in this study, it was 0.91.

#### 2.3.3. Self-Efficacy in Coping with Emergencies

I partially modified the self-efficacy scale developed by Jung et al. [32], as revised and supplemented by Lee [33], for suitability to the study context. After final revision and supplementation, the modified scale was reviewed, and its content validity analyzed by a group of experts (i.e., three nursing professors, two nursing team supervisors at OACFs, and two HCC directors). The modified 10-item tool is reported on a 10-point Likert scale, with total scores ranging from 10–100 points, and higher scores indicating advanced levels of self-efficacy. In Lee’s study, the Cronbach’s α was 0.95 [33]; in this study, it was 0.85.

#### 2.3.4. Confidence in Communication

A 5-item tool developed by Kim was used, which is reporteded on a numerical scale ranging from 0–10 points and has total scores ranging from 0–50 points [34]. In Kim’s study [34], the Cronbach’s α was 0.95; in this study, it was 0.89.

### 2.4. Research Process

**Figure 1:**
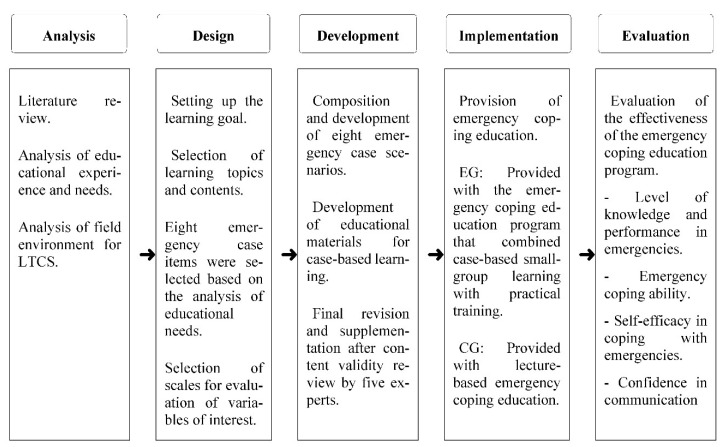
Development steps of the case-based small-group emergency coping education program for care workers. LTCS: long-term care services, EG: experimental group, CG: control group.

The education program was developed using the ADDIE model, a well-known instructional design system (Figure 1).

#### 2.4.1. Analysis

Prior to developing the case-based small-group emergency coping education program, a study on the “Comparison of emergency experience and first aid knowledge, emergency coping ability, educational experience, and educational needs of facilities and home caregivers” [13] was conducted. Further, a literature review was carried out to identify scientific evidence on care workers’ levels of emergency experience, first aid knowledge, emergency coping abilities, educational experience, and educational needs.

#### 2.4.2. Design

The learning contents and goals were defined based on the analysis of care workers’ educational needs. eight emergency case items were identified, some with high educational demand and some that were rarely experienced; in all cases, the non-provision of immediate aid could lead to permanent disabilities and/or fatal damage. Eight emergency cases were selected because they were both commonly experienced at LTCS facilities and require high levels of emergency coping abilities (e.g., airway obstruction and cardiac arrest). Specifically, they were airway obstruction (including dysphagia), dyspnea, cardiac arrest, stroke, loss of consciousness, falls, convulsions (seizures), and hypoglycemia.

#### 2.4.3. Development

The learning goals and scenarios were developed for each of the eight selected emergency cases, as shown in Table 1. The case scenarios were developed based on the real cases that are commonly experienced by care workers in the field. The learning contents comprised lesson plans, practical training plans, and performance evaluation sheets.

The lesson plans described the emergency case topic and the learning goals for each session. They comprised case contents, learning goals, motivations, learning activities, and finishing steps, which aimed to help care workers recall their own experiences and measures used to cope with similar emergencies.

The practical training was developed to enable care workers to simulate the act of reaching out for help upon recognizing an emergency and performing appropriate first aid measures until rescue team arrival.

The performance evaluation sheets allowed care workers to self-evaluate their emergency coping abilities, encouraged active participation in learning, and provided an opportunity for self-reflection.

As an educational aid for CBL, an emergency coping education program booklet for care workers was developed (as learning material) to help participants understand the case-based small-group learning activities, and practical training. The educational contents included the concept, causes, characteristics, and problematic symptoms related to each emergency scenario, how to cope with these situations, and precautions for emergency coping. The CBL materials and education booklet were used to ensure content validity, and the final revision and supplementation were conducted based on the results of a review by a group of experts (i.e., one emergency medicine doctor, two geriatric hospital doctors, two OACF or HCC directors, two nursing team supervisors, and three OACF or HCC care workers).

### 2.5. Implementation

#### 2.5.1. Emergency Coping Education Incorporating Case-Based Small-Group Learning

The case-based small-group emergency coping education program comprised eight modules (90 min per module), totalizing 12 h. The 12 h were divided into two 6-h educational sessions, which were implemented at one-week intervals.

Michaelsen et al. found that groups of six to eight people were effective for small-group learning [24]; accordingly, five small groups were formed, with seven to eight members each. The teams were decided by drawing lots, and the education program was implemented at the K University Simulation Center by the researcher and a gerontological nursing professor; both had experience in CBL. Each instructor was responsible for two sessions of two modules per day, totalizing four modules daily.

Prior to the first education session, the participants were provided with an orientation to the course and a 30 min introductory lecture on “case-based education for improving emergency coping abilities in care workers”.

In the introduction stage, cases related to the program contents for the current session were presented. To aid learning, media such as PowerPoint presentations and videos were used, and the care workers were encouraged to share their experiences and feelings about emergencies associated with older adults.

In the development stage, while in small groups, the participants learned activities 1, 2, and 3, and care workers were encouraged to actively communicate and interact with peers in the small group to identify measures to cope with the presented emergency.

Practical training sessions were provided at the simulation center. First, the professor demonstrated the first aid procedures for the given case, and then each care worker was given an opportunity to practice them. These practical sessions also included mock emergency situations, and each care worker was asked to perform the emergency measure according to the procedure described in the performance evaluation sheet for the case.

In the wrapping-up stage, the knowledge acquired through the learning process was evaluated. The care workers were asked to discuss the differences between their experience, the learning contents, and their feelings, all while interacting with their peers in the group.

#### 2.5.2. Lecture-Based Education

The researcher and a nursing professor provided lecture-based education for the CG using the same emergency education materials. It focused on eight emergency topics, lasted six hours per session, and was conducted over two sessions (totaling 12 h). The lectures were held in a seminar room using emergency education materials and videos.

### 2.6. Evaluation

To verify whether the developed program effectively improved care workers’ emergency coping abilities, their levels of knowledge and performance in emergencies, self-efficacy in coping with emergencies, and confidence in communication were evaluated.

### 2.7. Data Collection

Data were collected from 3–27 March 2021, using a structured self-reported questionnaire. In both the EG and CG, the pretest was conducted on the first day of the program, prior to the emergency coping education session. The posttest was conducted immediately after 12 h of course completion. Thus, the CG posttest data were collected around the same time the EG started the program. The questionnaire completion took approximately 30 min and they were collected immediately after completion to increase the collection rate.

### 2.8. Data Analysis

The collected data were analyzed using SPSS for Windows, version 25.0. Since this study comprised more than 30 participants, normality can be assumed by the central limit theorem. Data analysis was conducted following the procedures for χ^²^-test and independent *t*-test present in another study conducted with 57 nursing care workers [35], as follows:

- For the general characteristics of the EG and CG, percentages, means, and standard deviations were calculated.
- An independent *t*-test was performed for preliminary homogeneity analysis on the dependent variables of the two groups.
- The differences in the dependent variables between the two groups before and after the educational program were analyzed by an independent *t*-test.

### 2.9. Ethical Considerations

This study was reviewed and approved by the institutional review board of the researcher’s university (IRB No: SHIRB-202002-HR-104-03).

## 3. Results

### 3.1. Participants’ General Characteristics and Homogeneity Testing of the Two Groups

Care workers’ general characteristics, including their age, education level, work experience, working time, and workplace, demonstrated no statistically significant differences between the two groups. Additionally, no statistically significant differences were identified regarding participants’ initial levels of knowledge and performance in emergencies, emergency coping abilities, self-efficacy in coping with emergencies, and confidence in communication, thus confirming group homogeneity (Table 2).

### 3.2. Verification of the Difference in the Dependent Variable after Intervention

#### 3.2.1. Hypothesis 1

In the posttest, in the EG, the average level of knowledge in emergencies increased to 72.75 points; in the CG, it increased to 67.08, thereby indicating a statistically significant difference between the two groups (*t* = 4.379, *p* < 0.001). 

Furthermore, in the EG, the average level of performance in emergencies increased by 24.83 points; in the CG, it increased by 19.81 points, thus confirming the effect of the program between the two groups (*t* = 4.954, *p* < 0.001) and supporting Hypothesis 1 (Table 3).

#### 3.2.2. Hypothesis 2

In the posttest, in the EG, the average emergency coping abilities score increased by 30.75 points; in the CG, it increased by 26.80 points, which indicated a statistically significant difference between the two groups (*t* = 3.508, *p* < 0.001). 

Regarding subscales, in the EG, the basic life support performance score increased by 12.67 points; in the CG, it increased by 11.17 points, thereby indicating a statistically significant difference between the two groups (*t* = 2.855, *p* = 0.006). In the EG, the general first aid score increased by 18.08 points; in the CG, it increased by 15.63 points, thus confirming the difference between the two groups (*t* = 3.592, *p* = 0.001) and supporting Hypothesis 2 (Table 3).

#### 3.2.3. Hypothesis 3

In the posttest, in the EG, the average score for self-efficacy in coping with emergencies increased by 35.36 points; in the CG, it increased by 21.36 points, which indicated a significant difference between the two groups (*t* = 4.295, *p* < 0.001) and supported Hypothesis 3 (Table 3).

#### 3.2.4. Hypothesis 4

In the posttest, in the EG, the average score for confidence in communication increased by 14.42 points; in the CG, it increased by 12.01 points, thus indicating a significant difference between the two groups (*t* = 3.085, *p* < 0.003) and supporting Hypothesis 4 (Table 3).

### 3.3. Difference between Levels of Knowledge and Performance in Emergencies at Posttest

At posttest, in the EG, the average levels of knowledge and performance in emergencies were 3.64 and 3.67 points, respectively; although the performance level was slightly higher, no statistically significant difference was found. In the CG, the average levels of knowledge and performance of emergencies were 3.35 and 3.33 points, respectively, thereby indicating no statistically significant difference between the two variables at posttest (Table 4).

## 4. Discussion

Using a simulator, an emergency coping education program combining case-based small-group learning and practical training was developed and implemented, and its effectiveness at improving care workers’ emergency coping abilities was examined. Upon comparing the results for the EG and CG, a statistically significant difference was observed between the groups for the increase in the score for level of knowledge in emergencies at posttest, which supported the efficacy of the program for this variable. This result concurs with that of prior research, which has verified the effectiveness of case-based small-group learning and highlights that this type of learning is effective in expanding professional knowledge [23,36,37]. Since the educational contents of the developed program were grounded on real-life emergency situations that occurred among older adults in care workers’ workplace, the program allowed the participants to more comprehensively engage in active discussions and information sharing with peers. Moreover, self-reflection and the sharing of experiences are likely to have helped reinforce emergency knowledge among the participating care workers.

While there is a high demand for emergency education for care workers, they have insufficient availability of such educational opportunities. Moreover, the existing programs have often been problematic because they use lecture-based teaching methods, lack consideration for actual practice, and provide no practical training [18]. In this study, the learning was effective because learners were actively engaged in finding ideas and solutions to cope with the emergency, as the cases in the program reflected their daily practical experiences. The mutual teaching and learning that occurred owing to the free exchange of opinions and experience with colleagues broke the boundaries of conventional one-way lecture-based learning.

At posttest, in the EG the level of performance in emergencies was significantly higher than in the CG. This result finds symmetry in the literature, which applied CBL and observed improved clinical performance in participants [38,39]. Practical simulator-based training on first aid procedures is also known to enhance performance levels. In this study, each care worker had the chance to practice various procedures, step-by-step, while being thoroughly monitored by the researcher, and the feedback and suggestions provided to care workers during this process helped improve their performance. Combining CBL with practical simulation-based learning is an effective teaching strategy that has been shown to be capable of improving clinical performance [24]. This study recommends future emergency education for care workers to combine CBL with practical training.

Moreover, in the EG, the level of performance in emergencies increased more than the level of knowledge in emergencies; in the CG, the level of performance improved less than the level of knowledge. However, no statistically significant difference was identified in either group. To the best of my knowledge, no study has hitherto examined differences in the levels of knowledge and performance in emergencies. However, Park conducted a case-based cultural competency enhancement program for nurses, reporting that, at posttest in the EG, there were more cultural nursing behaviors than in the CG, albeit the differences were not statistically significant [40]. This finding corresponds to the results of the present study. On a related topic, Starr and Wallace stated that, although a short-term curriculum could change students’ knowledge or perceptions, it presents limitations regarding the ability to induce and sustain practical changes [41]. In Park’s study, the education program comprised six sessions, each of 90 min [40]; this format resembles the structure of thi study, in which a short-term educational program (i.e., six hours per day for two days, totalizing 12 h) was devised and implemented. Accordingly, neither Park’s study nor mine could induce long-term changes in performance; however, changes in the level of knowledge of emergencies were observed. Future studies should consider developing and implementing similar educational courses with a longitudinal approach and provide longitudinal assessments for the variables of interest.

In this study, the level of knowledge in the CG was higher than that of performance in emergencies. It is consistent with the results of Cho et al.’s study conducted with early childhood teachers, which reported a lower level of practice than of knowledge in emergencies related to children [30]. The findings are also consistent with another study that established that one-time lectures and observational education do not induce practical competency changes [42]. Furthermore, the findings support the results of prior research conducted with college students, which showed that an education program combining lectures and practice improved students’ intentions to perform and induced positive behavioral changes [43]. Therefore, considering that simulations can fill in gaps in practical skills among learners, maximize their learning (especially for difficult cases), and provide opportunities for self-reflection (through the debriefing process), it seems necessary to apply simulation education for care workers.

At pretest, in both the EG and CG the scores for emergency coping abilities were low (i.e., 55.06 and 43.97 points, respectively). Nevertheless, at posttest, both groups demonstrated great improvements in these coping abilities (EG, 85.81 points; CG, 78.58 points). Moreover, in the EG, the scores for the basic life support and general first aid subscales were significantly higher than in the CG. These results align with those of Prickett et al.’s study, which conducted an emergency tracheostomy education intervention for care workers; at posttest, care workers’ confidence in performance showed an increase [44]. Other studies that applied CBL have also observed an increase in the levels of practice performance [39,45,46], and the results support these earlier findings.

In dealing with older adult emergency situations, care workers must be capable of assessing the situation promptly and find appropriate solutions. In this study, through the delivery of eight different modules on different emergency cases, care workers were given opportunities to repeatedly practice first aid procedures for diverse emergency situations. Through indirectly engaging in the performance of emergency procedures, the developed program helped improve care workers’ problem-solving skills, thus enhancing their ability to cope with real-life emergencies. Although there is no evidence of the appropriateness of maintaining one’s emergency response capacity, It is still deemed that the cultivation of emergency coping abilities in care workers should be prioritized, as the life and prognosis of older adults are both dependent on care workers’ prompt and appropriate on-site responses in an emergency.

The analysis of pretest–posttest differences for self-efficacy in coping with emergencies between the two groups revealed significantly higher scores in the EG. This finding is in line with the results of previous studies that have applied case-based small-group learning [45,47,48]. In South Korea, care workers are most often the first emergency responders for LTCS recipients, which denotes that self-efficacy in coping with emergencies is a necessary skill for these professionals. In this study, the developed and implemented program seems to have been capable of increasing EG participants’ confidence in their ability to cope with emergencies, most possibly owing to being repeatedly exposed to case-based education and practical training on emergency scenarios. Moreover, the small-group learning approach provided opportunities for participants to correct their misconceptions on topics surrounding emergency situations. According to Dykes et al., care providers with high self-efficacy perform their work zealously in order to prevent health problems among older adults, and higher levels of self-confidence result in advanced levels of relevant knowledge and skills [49]. Since CBL has been evaluated as a useful teaching strategy to enhance care workers’ self-efficacy in coping with emergencies [45,50]. The active use of the emergency coping education program proposed in this paper for care workers in South Korea is recommended.

At posttest, in the EG the score increase for confidence in communication was significantly higher than in the CG, thus indicating the program’s effectiveness in improving this variable. This finding is in line with that of Choi’s study, which conducted a shared mental model-based emergency management training program for workers in OACF and showed a significant increase in the communication confidence of the EG [51]. It also supports the results of previous studies that have reported that CBL enhances communication confidence [34,52,53,54]. Still, Choi remarked that no studies, thus far, have explored the role of communication among practitioners managing emergencies at LTCS sites [51]. Despite this drawback, a study showed that when an emergency occurs, it is common for practitioners to cope with the situation by interacting with one another through communication and information sharing [55]. Therefore, it is highly likely that smooth communication will greatly and positively impact emergency management in practice. Of note, research reveals that 80% of medical accidents result from inappropriate communication among healthcare workers [56]. Therefore, workers who may face emergencies should have adequate communication skills to report emergencies successfully [57].

In this study, learning activity 1 of the education program aimed to reinforce care workers’ knowledge of emergencies, while learning activity 2 provided practical, step-by-step training to recognize emergencies, on the reporting system, and on the first aid treatment that should be administered until the arrival of the rescue team. De Meester et al. stated that, when faced with an emergency, especially in the clinical/practical field, even experienced practitioners might feel uncertain about their judgments and fear making an incorrect emergency report [54]. Hence, providing an education booklet containing content on each emergency (which the educational program of the current study did) may increase care workers’ self-confidence in their communication skills and performance during emergencies. Moreover, educational programs must have a standardized format to improve communication, which should be characterized by the concise delivery of extensive information within a limited time [58]. Coupled with the current research results, It is posited that systematic education and training can develop care workers’ communication skills.

The study results showcased how the program development and implementation had a positive impact on care workers’ emergency coping abilities. Nonetheless, the CG, which was provided with lecture-based education, also showed improvements on all variables at posttest. These results may be related to the fact that this lecture-based program had materials and videos that were tailored for care workers, and it was implemented against a backdrop of a scarcity of opportunities for receiving systematic emergency education. However, owing to the absence of practical training in the CG, their level of performance in emergencies showed no improvements at posttest. This result suggests the key role of practical training in emergency education. In summary, in order to strengthen care workers’ emergency coping abilities, stakeholders in South Korea should provide all such professionals with the opportunity to regularly engage in emergency education, thereby denoting the pressing need of institutionalizing refresher training and on-the-job education systems.

While the results provide a number of meaningful insights, this study has some limitations as well. First, since the participants were selected through convenience sampling from workers at OACFs and HCCs in specific regions, generalizations should be made with caution. Second, the level of performance in emergencies was measured at posttest with a self-reported questionnaire, which denoted that participants’ actual levels of performance may differ from the current, self-reported results. Future research should use direct measurement tools for this construct at posttest. Third, prior research shows that the measurement of the effects of an educational program at posttest should be carried out at least two weeks after the period required for learning transition [59]. However, owing to the national distancing policy related to the COVID-19 pandemic that was active at the time of this study, it was difficult to conduct a posttest assessment after the occurrence of learning transfer. Accordingly, the assessment had to be conducted immediately after the end of the program. Future research should measure the effect of the program while considering the time required for learning transfer to occur. Fourth, this study did not reflect the effect of education related to the experience of care workers because it did not measure whether the experience of care workers in emergency situations actually affected the improvement of emergency knowledge.

## 5. Conclusions

With the rapid aging population phenomenon in South Korea, the number of recipients of the LTC insurance has increased by 15.1% compared to the previous year. This study is significant in that it developed, implemented, and examined the effects of a case-based emergency coping education program [37].

Herein are the implications of this study for professional nursing education. First, since the proposed program had a positive effect on care workers’ emergency coping abilities, its contents can be used as data for job training and maintenance training aimed at improving care workers’ practical skills in emergency situations. It is also expected that this program could indirectly contribute to improving the survival rate and reduce medical expenses in South Korean older adults by preventing health complications. Second, stakeholders could use the program contents as a framework for the development of practical guidelines for handling emergency situations, which can then be disseminated to service sites. This provision may enable care workers to quickly respond to changes in older adults’ health status, improve safety management for older adults, and reduce the risk of older adults to incur health complications. These risk minimization efforts may help improve the quality of the LTCS.

Herein are the implications of this study for the literature. Given that the program had its educational effectiveness demonstrated, to maximize the learning effect of the program, future research should develop and apply an education program that combines the case-based small-group learning method with simulation education. Second, the short-term program’s effect after 12 h of its completion were measured; hence, future studies should measure the educational effects of longitudinal education programs with a large sample size. Third, further research on how to longitudinally sustain care workers’ levels of knowledge and performance in an emergency is suggested, which can be operationalized by the continuous development and effectiveness verification of various emergency response training plans centered on systematic and enhanced case studies.

## Figures and Tables

**Table 1:** Contents of the case-based small-group emergency coping education program.

Modules	Learning Objectives	Lesson Contents	Time (min)	Pedagogy Strategy
(Introduction) Case-based education for improving emergency coping ability in care workers	Understanding the purpose and goal of case-based education	Case-based education to improve emergency coping ability Orientation of program process and objectives Introduction to lesson schedule and contents Sharing experiences of emergency situations associated with older adults Introduction to future lesson content and schedule Organization of small groups Conducting pretest	60	Communication Small-group learning Interactions among learners Self-reflection Questions Lecture
(Module 1) Older adults whose heart stopped beating	Understanding the concept and signs of cardiac arrest Learning about the causes and symptoms of cardiac arrest Understanding how to cope with cardiac arrest, in case of an emergency Performing emergency measures according to the procedures for cardiac arrest	Sharing experiences of the previous lesson and introduction to the current lesson schedule Learning the definition, causes, and warning symptoms of cardiac arrest, as well as understanding how to check for cardiac arrest Learning how to cope with cardiac arrest, in case of an emergency Learning about the basic life support procedures and methods	50	Communication Small-group learning Self-reflection Interactions among learners Questions and feedback Problem-solving
		Practicing the emergency measures for cardiac arrest Practicing basic life support procedures Introduction to future lesson content and schedule	40	Demonstration Practice
(Module 2) Older adults with a rice cake stuck in the throat	Understanding the concept of airway obstruction and how to recognize it Learning about the causes and symptoms of airway obstruction Understanding how to cope with airway obstruction, in case of an emergency Performing emergency measures according to the procedures for airway obstruction	Sharing experiences of the previous lesson and introduction to the current lesson schedule Understanding the causes, symptoms, and treatment of airway obstruction owing to a foreign body Understanding how to cope with each type of airway obstruction, in case of an emergency Learning about the structure of the human airway Learning about the Heimlich maneuver procedures and methods	50	Communication Small-group learning Self-reflection Interactions among learners Questions and feedback Problem-solving
		Practicing the emergency measures for airway obstruction Learning about the Heimlich maneuver procedures and methods Learning about the Heimlich maneuver procedures and methods for each type of airway obstruction Introduction of future lesson content and schedule	40	Demonstration Practice
(Module 3) Older adults showing signs of reduced brain function	Understanding the concept and prodromes of stroke Learning about the causes and symptoms of stroke Understanding how to cope with stroke, in case of an emergency Performing emergency measures according to the procedures for stroke	Sharing experiences of the previous lesson and introduction to the lesson schedule Learning the definition, causes, prodromes, and symptoms of stroke Understanding how to cope with stroke, in case of an emergency	50	Communication Small-group learning Self-reflection Interactions among learners Questions and feedback Problem-solving
		Practicing the emergency measures for stroke Introduction to future lesson contents and schedule	40	Demonstration Practice
(Module 4) Older adults falling unconscious	Understanding the concept of loss of consciousness Learning about the causes and symptoms of loss of consciousness Understanding how to cope with loss of consciousness, in case of an emergency Performing emergency measures according to the procedures for loss of consciousness	Sharing experiences of the previous lesson and introduction to the current lesson schedule Learning the definition and causes of loss of consciousness Learning about the procedures and methods of checking for loss of consciousness Learning about the problems associated with loss of consciousness Learning about the emergency measures for loss of consciousness	50	Communication Small-group learning Self-reflection Interactions among Learners Questions and feedback Problem-solving
		Practicing the methods of checking for consciousness Practicing the emergency measures for loss of consciousness Introduction to future lesson contents and schedule	40	Demonstration Practice
(Module 5) Older adults suffering from shortness of breath	Understanding the concept of dyspnea Learning about the causes and symptoms of dyspnea Understanding how to cope with dyspnea, in case of an emergency Performing emergency measures according to the procedures for dyspnea	Sharing experiences of the previous lesson and introduction to the current lesson schedule Learning about the definition, causes, and symptoms associated with dyspnea Learning how to cope with hypoglycemia, in case of dyspnea	50	Communication Small-group learning Self-reflection Interactions among learners Questions and feedback Problem-solving
		Practicing the emergency measures for dyspnea Introduction to future lesson content and schedule	40	Demonstration Practice
(Module 6) Older adults having a severe convulsion (seizure)	Understanding the concept and prodromes of convulsion (seizure) Learning about the causes and symptoms of convulsion (seizure) Understanding how to cope with severe convulsion (seizure), in case of an emergency Performing emergency measures according to the procedures for convulsion/seizure	Sharing experiences of the previous lesson and introduction to the current lesson schedule Learning the definition, causes, prodromes, and symptoms of convulsion (seizure) Learning how to cope with convulsion (seizure), in case of an emergency	50	Communication Small-group learning Self-reflection Interactions among learners Questions and feedback Problem-solving
		Practicing the emergency measures for prodromes Introduction to future lesson content and schedule	40	Demonstration Practice
(Module 7) Older adults feeling dizzy owing to low blood sugar	Understanding the concept of hypoglycemia Learning about the causes, prodromes, and symptoms of hypoglycemia Understanding how to cope with hypoglycemia, in case of an emergency Performing emergency measures according to the procedures for dealing with hypoglycemia	Sharing experiences of the previous lesson and introduction to the current lesson schedule Learning the definition, causes, and symptoms of hypoglycemia Learning how to cope with hypoglycemia, in case of an emergency	50	Communication Small-group learning Self-reflection Interactions among learners Questions and feedback Problem-solving
		Practicing the emergency measures for hypoglycemia Introduction of future lesson content and schedule	40	Demonstration Practice
(Module 8) Older adults falling in the living room and being unable to move	Understanding the concept of a fall Learning about the causes of a fall Learning how to check for and how to cope with damage, in case of an emergency Performing emergency measures according to the procedures for dealing with a fall	Sharing experiences of the previous lesson and introduction to the current lesson schedule Learning the definition, causes, and symptoms of a fall Learning how to cope with a fall, in case of an emergency	50	Communication Small-group learning Self-reflection Interactions among learners Questions and feedback Problem-solving
		Practicing the emergency measures for a fall Wrapping-up of the program and evaluation Conducting posttest	40	Demonstration Practice

**Table 2:** Homogeneity test of general characteristics and outcome variables between the experimental and control groups (*N* = 72).

Variables	Categories	Experimental Group (*n* = 36) *n* (%) or M ± SD	Control Group (*n* = 36) *n* (%) or M ± SD	χ^2^*/t*	*p*
Age (yr)	Average age	60.47 ± 4.48	59.78 ± 4.85	0.631	0.530
Education	Middle school or less	7 (19.4)	9 (25.0)	0.444	0.910
	High school	27 (75.0)	24 (66.7)		
	College or above	2 (5.6)	3 (8.3)		
Work experience	Less than 1 year	1 (2.8)	4 (11.1)	3.050	0.569
	1 to less than 3 years	7 (19.4)	5 (13.9)		
	3 to less than 5 years	7 (19.4)	8 (22.2)		
	5 to less than 10 years	8 (22.2)	10 (27.8)		
	Over 10 years	13 (36.1)	9 (25.0)		
Working time	Everyday 9 to 6	7 (11.1)	8 (22.2)	0.508	0.776
	Three shifts	11 (38.9)	8 (22.2)		
	Part-time	18 (50.0)	20 (55.6)		
Work place	OACF	18 (50.0)	16 (44.4)	0.223	0.637
	HCC	18 (50.0)	20 (55.6)		
Outcome variables					
Emergency situation	Knowledge level	51.56 ± 10.57	48.33 ± 3.58	1.733	0.090
	Practice level	48.50 ± 5.42	46.75 ± 3.77	1.590	0.116
Emergency coping ability	Basic life support	21.42 ± 3.51	20.33 ± 3.66	1.281	0.204
	General first aid	33.64 ± 5.87	31.44 ± 3.26	1.961	0.054
	Total	55.06 ± 8.86	51.78 ± 5.52	1.884	0.064
Self-efficacy in coping with emergencies		50.17 ± 10.56	48.06 ± 3.64	1.134	0.263
Confidence in communication		19.39 ± 5.63	17.06 ± 5.70	1.747	0.085

OACF: older adult care facility, HCC: home care center.

**Table 3:** Pretest-posttest differences by the experimental and control groups (*N* = 72).

Variables		Groups	Pretest	Posttest	Difference	*t*	*p*
			Mean ± SD	Mean ± SD	Mean ± SD		
Emergency situation	Knowledge level	EG (*n* = 36)	51.56 ± 10.57	72.75 ± 4.81	21.19 ± 13.08	4.379	<0.001 *
		CG (*n* = 36)	42.78 ± 4.99	67.08 ± 6.10	18.75 ± 6.52		
	Performance level	EG (*n* = 36)	48.50 ± 5.42	73.33 ± 3.62	24.83 ± 6.71	4.954	<0.001 *
		CG (*n* = 36)	39.81 ± 5.06	66.56 ± 7.37	19.81 ± 7.07		
Emergency coping ability	Basic life support	EG (*n* = 36)	21.42 ± 3.51	34.08 ± 3.23	12.67 ± 4.34	2.855	0.006 *
		CG (*n* = 36)	17.56 ± 1.50	31.50 ± 4.37	11.17 ± 6.75		
	General first aid	EG (*n* = 36)	33.64 ± 5.87	51.72 ± 4.08	18.08 ± 6.39	3.592	0.001 *
		CG (*n* = 36)	26.42 ± 3.40	47.08 ± 6.59	15.63 ± 8.87		
	Total	EG (*n* = 36)	55.06 ± 8.86	85.81 ± 6.76	30.75 ± 12.72	3.508	0.001 *
		CG (*n* = 36)	43.97 ± 4.41	78.58 ± 10.34	26.80 ± 14.43		
Self-efficacy in coping with emergencies		EG (*n* = 36)	50.17 ± 10.56	85.53 ± 12.71	35.36 ± 17.21	4.295	<0.001 *
		CG (*n* = 36)	43.72 ± 10.82	69.42 ± 18.58	21.36 ± 17.17		
Confidence in communication		EG (*n* = 36)	19.39 ± 5.63	33.81 ± 5.82	14.42 ± 7.73	3.085	0.003
		CG (*n* = 36)	17.06 ± 5.70	29.08 ± 7.11	12.01 ± 9.34		

*p* = 0.05, EG: experimental group, CG: control group.

**Table 4:** Differences between levels of knowledge and performance in emergencies at posttest (*N* = 72).

Variables		Groups	Knowledge Level	Performance Level	Means Difference	*t*	*p*
			Mean ± SD	Mean ± SD			
Differences between levels of knowledge and performance	Total	EG (*n* = 36)	3.64 ± 0.24	3.67 ± 0.18	0.03	−0.627	0.535
		CG (*n* = 36)	3.35 ± 0.30	3.33 ± 0.37	0.02	0.461	0.648

*p* = 0.05, EG: experimental group, CG: control group.
